# Synthesis and Characterization of ZIF-90 Nanoparticles as Potential Brain Cancer Therapy

**DOI:** 10.3390/pharmaceutics16030414

**Published:** 2024-03-18

**Authors:** Lorenzo Monarca, Francesco Ragonese, Paola Sabbatini, Concetta Caglioti, Matteo Stamegna, Federico Palazzetti, Paolo Sportoletti, Ferdinando Costantino, Bernard Fioretti

**Affiliations:** 1 Department of Chemistry, Biology and Biotechnologies, University of Perugia, Via dell’Elce di Sotto 8, 06123 Perugia, Italy; francesco.ragonese@unipg.it (F.R.); paola.sabbatini@unipg.it (P.S.); concetta.caglioti@studenti.unipg.it (C.C.); matteo.stamegna@studenti.unipg.it (M.S.); federico.palazzetti@unipg.it (F.P.); 2 Department of Experimental Medicine, Perugia Medical School, University of Perugia, 06132 Perugia, Italy; 3 Department of Medicine and Surgery, Institute of Hematology, Centro di Ricerca Emato-Oncologica (CREO), University of Perugia, 06129 Perugia, Italy; paolo.sportoletti@unipg.it

**Keywords:** glioblastoma, U251, nanoparticles, MOF, ZIF-90, drug targeting, berberine

## Abstract

Human glioblastoma is probably the most malignant and aggressive among cerebral tumors, of which it represents approximately 80% of the reported cases, with an overall survival rate that is quite low. Current therapies include surgery, chemotherapy, and radiotherapy, with associated consistent side effects and low efficacy. The hardness in reaching the site of action, and overcoming the blood–brain barrier, is a major limitation of pharmacological treatments. In this paper, we report the synthesis and characterization of ZIF-90 (ZIF, Zeolitic Imidazolate Framework) nanoparticles as putative carriers of anticancer drugs to the brain. In particular, we successfully evaluated the biocompatibility of these nanoparticles, their stability in body fluids, and their ability to uptake in U251 human glioblastoma cell lines. Furthermore, we managed to synthesize ZIF-90 particles loaded with berberine, an alkaloid reported as a possible effective adjuvant in the treatment of glioblastoma. These findings could suggest ZIF-90 as a possible new strategy for brain cancer therapy and to study the physiological processes present in the central nervous system.

## 1. Introduction

Glioblastoma is a malignant astrocytic tumor, considered the most dangerous among brain tumors in adults, with an annual incidence that ranges from 0.59 to 3.69 per 100,000 persons and a prevalence 1.5 fold higher in men compared with women [1] that is rising in many countries [2]. Glioblastoma can occur at any age, but in 70% of cases, it is diagnosed in patients aged between 45 and 70 years. Tumors are usually located in the cerebral hemispheres but can occur throughout the central nervous system. Primary glioblastoma has a rapid and progressive course (around 2–3 months), except when it develops from a pre-existing low-grade astrocytoma (secondary glioblastoma) [3].

Current glioblastoma therapy consists of a partial or complete surgical resection that can only be performed rarely, considering that tumor cells usually infiltrate the surrounding brain areas [4]. The treatment is integrated with radiotherapy targeted to the tumor bed, in combination with chemotherapy [5,6]. Adjuvant treatment after surgery can improve overall survival, for example, with monoclonal antibodies [7], but further investigations are still required to understand the mechanism and the efficacy of these approaches [5].

The main obstacle to the diagnosis and treatment of glioblastoma is the blood–brain barrier (BBB), which prevents more than 98% of contrast agents or therapeutic drugs from reaching the brain [8,9,10,11]. Therefore, a high dose of medication is usually required to treat glioblastoma, which often causes serious side effects [6]. Furthermore, therapeutic efficacy is limited by the high level of drug resistance of glioblastoma cells, which leads to an unfavorable prognosis and high relapse rates, with mutations of the tumor cells, both locally and distantly, from the site of primary origin of the cancer. Distant recurrences typically share only an average of 25% of mutations with their primary tumors compared with an average of 70% of mutations shared in local recurrences [12]. 

Interesting results can be obtained using nanoparticles. For their dimension they are able to cross the BBB, diffusing specifically in the leaky vasculature of the tumor area; therefore, nanoparticles are promising structures to realize effective nanocarriers [13]. Among the possible compounds, recently, the possible use of Metal–Organic Frameworks (MOFs) in bio- and nanomedicine is gaining attention [14,15]. MOFs are inorganic–organic hybrid compounds with porous crystalline structures constituted of polynuclear metal clusters (also called nodes) linked to each other by organic ligands such as carboxylates, phosphonates, heterocycles, and so on. Their inherent properties such as ordered and tunable porosity, good crystallinity, and high surface areas make them suitable for a large range of applications. MOFs find use in catalysis [16], carbon dioxide adsorption [17], energy storage [18], natural gas purification [19], battery optimization [20], and many other fields. The versatility of MOFs is related to the option to select the most suitable linker or metal, to modulate their dimension, and to support post-synthetic modifications, for instance, biomolecule conjugation [21]. The application in nanomedicine is suggested by the possibility of using highly biocompatible metals (id est, Fe, Zn, or Zr) and/or linkers [22]. In particular, the possibility of using MOFs as drug carriers, bioimaging agents, and therapeutic agents themselves is under investigation [23]. 

In this field, we previously reported the synthesis and characterization of ultrasmall UiO-66_NP (Universitetet i Oslo) MOF based on a [Zr_6_O_4_(OH)_4_] hexanuclear cluster combined with 1,4-benzodicarboxylic acid. These MOF nanoparticles, without any surface modification, were internalized in U251 cells, a permanent cell line derived from a human malignant glioblastoma multiforme, and retained within them, without inducing morphological changes or decrease in cell viability [24].

Recently, Sharma et al. developed a MOF loaded with two potential anticancer agents, cannabidiol and gallic acid, to treat rat glioma in brain cancer cell line (C6). The slow degradation of cannabidiol/magnesium gallate MOF resulted in a promising reduction in the proliferation of cancer cells. The anti-tumor effects of cannabidiol target mitochondria, inducing a significant increase in ROS production and a reduction in anti-inflammatory responses as reflected by a significant decrease in Tumor Necrosis Factor α (TNF-α) expression levels. Molecular mechanisms underlying these effects include the modulation of nuclear factor kappa-light-chain-enhancer of activated B cells (NF-kB) expression, triggering apoptotic cascades of glioma cells [25]. 

Prompted by these interesting preliminary results, we decided to expand our studies on the development of MOFs for the treatment of glioblastoma, in particular, using ZIF-90 (Zeolitic Imidazolate Framework 90), which seems promising in the field of drug delivery, with excellent thermal and chemical stability, permanent porosity, and uniform pore size. ZIF structures are composed of a very biocompatible and biosafe metal (zinc) [26] and an organic linker (ICA, imidazole-2-carboxaldehyde), which is feasible for further modifications, such as oxidation and drug or antibody conjugation [27]. ZIF-90 has caught our attention because of the high biocompatibility of zinc, as well as its ability to make further modifications, such as protein conjugation, owing to its exposed aldehyde groups. Moreover, ZIFs have proven to be able to overcome the BBB in mouse models [28]. The mechanism of this process is currently under investigation. Recently, Jiang et al. suggested that the passage occurs by clathrin-mediated and caveolae-mediated endocytosis [29].

In this work, we tried to verify the potential of ZIF-90 as a nanocarrier for the treatment of glioblastoma, using human glioblastoma cell line U251 as a cellular model. The biocompatibility of MOFs synthesized in an aqueous system was evaluated through specific methodologies; furthermore, ZIF-90 loaded with acridine orange (AO) was synthesized to trace the position of the drug after cellular absorption. As a further investigation, we explored the potential of ZIF-90 to encapsulate and release drugs into target cells to implement therapies against glioblastoma. Natural compounds represent an important opportunity to develop a new therapeutic approach against glioblastoma. Recently, the use of nutraceutical compounds as adjuvant therapy has been proposed, such as resveratrol [30] and berberine [31]. In particular, for our study, we selected berberine, a benzylisoquinoline alkaloid extracted from roots, rhizomes, or bark of several plants, mostly belonging to the *Berberis* genus, like *B. vulgaris* (barberry), but also in other plants such as *Hydrastis canadensis* or *goldenseal* and *Coptis chinensis* (Chinese goldthread). Modern medicine has discovered this molecule for its application in a wide range of diseases, showing anti-inflammatory, antioxidant, antidiabetic, antihyperlipidaemic, antiobesity, cardioprotective, and anticancer properties [32]. 

Many effects of berberine pharmacology have been demonstrated to be linked to its effect on gut microbiota [33], but berberine can also affect numerous signaling pathways, including the phosphatidylinositol 3-kinase/protein kinase B (PI3K/Akt), NF-κB, the adenosine monophosphate-activated protein kinase (AMPK), the cyclic-AMP response element-binding protein (CREB), the nuclear factor erythroid 2–related factor 2 (Nrf2), and the mitogen-activated protein kinase (MAPK) pathways, thus acquiring neuroprotective effects [34]. Recently, it has been reported that berberine can inhibit the TGF-β1/SMAD2/3 signaling pathway, thus suppressing tumor progression in four human glioma cell lines [35].

It is reported that berberine has a very low bioavailability (less than 1% of the ingested amount is actually absorbed by the gut) [36]; moreover, a great amount of berberine absorbed by the gut is then catabolized by liver first-pass (in phase I, it is metabolized by demethylation, and in phase II, by glucuronidation) elimination [37]. Unsurprisingly, the bioavailability increases by avoiding the gastric/intestinal route, id est, after intraperitoneal injection [38]. Regardless of the low plasma concentration, berberine accumulates in most organs and tissues, especially in the liver, and it is rapidly excreted via the urine [39]. 

The loading of berberine into nanocarriers may improve its bioavailability and its accumulation in specific target tissues. Recently, berberine encapsulated in poly(lactic-co-glycolic acid) (PLGA)-based nanoparticles was reported to reduce the viability of established T98G glioblastoma cells by about 50% [40]. Other strategies could be developed to increase berberine’s pharmacokinetic profile. For example, we report the increase in bioavailability of resveratrol, a molecule with low solubility, by supporting it via an inorganic matrix, such as magnesium dihydroxide [41,42]. In the last part of this work, therefore, we report the preparation and characterization of berberine-loaded ZIF-90 nanoparticles as a potential delivery strategy and treatment of glioblastoma.

## 2. Materials and Methods

### 2.1. Materials

Reagents and solvents from commercial suppliers (Sigma Aldrich, St. Louis, MO, USA) were used directly without further purification. For cell culture, Dulbecco’s Modified Eagle Medium/Nutrient Mixture (DMEM), fetal bovine serum (FBS), penicillin G, and streptomycin were purchased from EuroClone S.p.A. (Pero, Milan, Italy), and sterile flasks were purchased from Falcon, Corning (Glendale, Arizona, USA). The U251 and HEK293 cell lines were obtained from Cell Lines Service GmbH (Eppelheim, Heidelberg, Germany). The HeLa cell line was a gift from Professor Cataldo Arcuri (Department of Medicine and Surgery, University of Perugia).

### 2.2. ZIF-90, ZIF-90@Acr, and ZIF-90@Berb Synthesis

The synthesis of ZIF-90 nanoparticles was carried out according to the literature [43]. Briefly, two different solutions, A (water and glycerol 1:1 in volume ratio) and B (water and tert-butanol 1:1 in volume ratio), were prepared separately. In solution A, ICA was dissolved under stirring and warming conditions; zinc nitrate was instead dissolved in B solution. Synthesis of ZIF-90 was obtained by mixing, under vigorous stirring, A and B in a 1:1 ratio. The microcrystalline powder was then collected by centrifugation and washed with methanol and acetone. In order to encapsulate other molecules inside the ZIF-90 pores, the one-pot synthesis method is most suitable. Berberine or AO (1:100 molar ratio with ICA) was dissolved in the B solution before adding the A solution, obtaining ZIF-90@Berb or ZIF-90@Acr, respectively.

### 2.3. X-ray Powder Diffraction (XRPD) Analysis

The XRPD patterns were collected in the 3–60° 2θ range and with a 40 s/step counting time with the CuKα radiation on a PANalytical X’PERT PRO diffractometer (Malvern Panalytical Ltd., Worcestershire, UK), PW3050 goniometer (Malvern Panalytical Ltd., Worcestershire, UK), equipped with an X’Celerator detector (Malvern Panalytical Ltd., Worcestershire, UK). The long fine focus (LFF) ceramic tube operated at 40 kV and 40 mA.

### 2.4. Electron Microscopy Analysis

Scanning electron microscopy (SEM) images were obtained with a LEO 1525 (Zeiss, Jena, Germany) microscope equipped with a Gemini column. Transmission electron microscopy (TEM) images were obtained with a Philips EM 400 microscope.

### 2.5. Gas Sorption Measurements

A Micromeritics 2010 apparatus (Micromeritics, Norcross, GA, USA) was used to obtain the adsorption and desorption isotherms with nitrogen at 77 K. Before the adsorption analysis, the samples were first soaked in chloroform for two days. Then, they were outgassed at 100 °C under a vacuum overnight before starting the measurement. The introduction of low boiling point polar solvents such as chloroform before absorption analysis helps the degassing process before BET analysis [44,45].

### 2.6. UV–Vis Spectroscopy

Spectra in the UV–Vis absorbance range were obtained with a Varian Cary 100 Scan and elaborated with Origin 6.1 software. ZIF-90 was dissolved at 10 μg/mL in distilled water and measured directly in a quartz cuvette. The absorbance baseline was measured on solvent alone.

### 2.7. Thermogravimetric (TG) Analysis

TG measurements on ZIF-90 and ZIF-90@Acr powder were performed using a Netzsch STA490C thermoanalyzer (NETZSCH Group) under a 20 mL/min air flux with a heating rate of 10 °C/min.

### 2.8. High-Performance Liquid Chromatography (HPLC) Analysis

The mass amount of encapsulated berberine was determined with a Varian ProStar 210 HPLC chromatograph equipped with a Gemini (Phenomenex, Torrance, CA, USA) 5 µm C18 110 Å, LC Column 150 mm × 4.6 mm, and a UV–vis detector. The mobile phase was composed of 30% of 0.05% formic acid in methanol (HPLC grade, Sigma Aldrich) and 70% of 0.05% formic acid in acetonitrile (HPLC grade, Sigma Aldrich) at 1 mL/min flow rate. The measurements were taken at 260 nm.

Standard solutions for the calibration curve were prepared by dissolving the standard berberine hydrochloride in methanol to obtain the following concentrations: 2.98 µg/mL; 5.96 µg/mL; 11.9 µg/mL; 23.8 µg/mL; and 47.7 µg/mL (see Supplementary Materials). 

To extract berberine from the ZIFs, about 1–2 g of ZIF was weighted and 10 mL of methanol was added. The mixture was vortexed for 15 s and centrifugated for 15 min at 4.500 g. The supernatant was collected in a 50 mL Falcon tube; then, 10 mL of methanol was added to the pellet, which was vortexed for 15 s and centrifugated for 15 min at 4500 g again. The second supernatant was added to the previously collected one in the 50 mL Falcon tube and vortexed for an additional 15 s. An amount of 20 µL of the obtained solution was diluted with 980 µL of methanol and injected into the HPLC column.

### 2.9. Cell Culture

Cells (U251, HEK293, and HeLa) were grown in DMEM with high glucose supplemented with 10% FBS, 100 IU/mL penicillin G, and 100 µg/mL of streptomycin in H_2_O-saturated 5% CO_2_ atmosphere at 37 °C and in sterile flasks. Cell confluence was held strictly below 70%, if not differently specified.

### 2.10. Internalization of ZIF-90 Evaluation 

U251 cells were seeded the day before treatment in 35 mm Petri dish plates (100,000 cells per plate). An amount of 50 μL of ZIF-90@Acr (or ZIF-90 as negative control) 1 mg/mL solution was added to the cells the day of the experiment. At certain time points, plates were intensively washed in order to remove non-internalized nanoparticles and were observed by microscope. Internalization was evaluated with a fluorescence microscope (AxioExaminer, Zeiss, Jena, Germany) as fluorescence intensity due to AO presence. Data are presented as the mean of single cell measurements (n = 30) ± SE.

### 2.11. Stability of ZIF-90 in Simulated Body Fluid (SBF) Evaluation

ZIF-90 was dissolved at a rate of 1 mg/mL in 10 mL of sterile DMEM and poured into a dialysis tube. SBF was established as DMEM with low glucose at 37 °C and continuously under vigorous stirring. The dialysis tube was fixed in a beaker with SBF. At precise time points, 1 mL of external solution was taken and analyzed using inductively coupled plasma mass spectrometry (ICP-MS) (Thermo Scientific, Waltham, MA, USA, X SERIES ICP-MS equipped with NEW WAVE UP213 Solid State Laser Ablation system). The absolute value was corrected considering the change in volume after every draw. Data are presented as mean of independent measures ± SE.

### 2.12. Cellular Vitality Assay

U251, HEK293, or HeLa cells were seeded in 96-well plates (Falcon, Corning) with a cell density of 3000 cells for the well. After 24 h, cells were treated with different concentrations of ZIF-90 for 48 h. For viability tests, MTT (3-(4,5-dimethylthiazol-2-yl)-2,5-diphenyltetrazolium bromide tetrazolium) reduction colorimetric assay (Sigma Aldrich) was used. For the assay, cells were incubated with MTT at a final concentration of 0.5 mg/mL for 3 h at 37 °C. The medium was removed and insoluble formazan crystals remained adhered to the bottom of the wells. After solubilization in dimethyl-sulfoxide, the formazan dye was quantified using a Varian Cary 100 scan spectrophotometer (Agilent, Santa Clara, CA, USA). The experimental data were expressed as the mean of the recorded values normalized for the sample with 0 ng/mL of ZIF-90 (±SE).

### 2.13. Chick Embryo Chorioallantoic Membrane (CAM) Assay

An irritation CAM assay was performed according to the literature [46]. Briefly, fertilized eggs (Azienda Agraria Cerquaglia S.s. Agricola di Elisabetta e Stefano Cerquaglia) were incubated at 37.2 °C in an atmosphere saturated with water, with the egg bladder facing up; unfertilized eggs or unhealthy embryos were rejected. After two days, the top part of the eggshell was excised and 5 mL of albumen was removed; then, the egg was sealed with parafilm and re-incubated for two more days, until CAM was vascularized enough to be tested. Then, the egg was mounted under a stereomicroscope (Axiozoom V16, Zeiss, Jena, Germany) and 1 mL of the testing solution (ZIF-90 1 mg/mL, PBS or SDS 0.5% as the negative and positive controls, respectively) was dropped on the CAM. A photo was taken at 0.5, 2, and 5 min after the treatment according to the literature. In these three photos, the appearance of hyperemia, hemorrhage, and coagulation hints were observed, and a certain score, from 0 to 21 points, was given depending on the time of first observation.

### 2.14. Statistical Analysis

OriginLab software Origin 6.1 was used for the statistical analysis. Each result is the average of three independent experiments, reported with relative standard errors and relative statistical significance (*p* < 0.05) obtained using Student’s *t*-test.

## 3. Results

**Figure 1:**
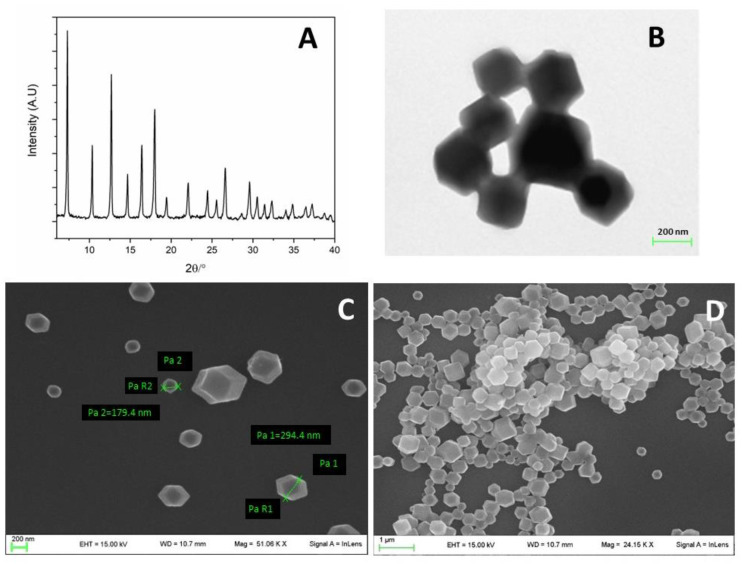
Morphological characterization of ZIF-90. (**A**) XRPD pattern of ZIF-90. (**B**) TEM photo of ZIF-90. (**C**,**D**) SEM photos of ZIF-90.

ZIF-90 has caught our attention for its high biocompatibility and biosafety of zinc, and because of the option to perform further modifications, such as protein conjugation, owing to its exposed carbonyl group [47]. ZIF-90 nanocrystals were obtained as described in Section 2. The synthesis was successfully confirmed by X-ray powder (XRPD) analysis (Figure 1A): all the peaks, especially the most evident at 3.5, 5.5, 6.5, and 7 2θ degrees, were coincident with the reported values in the literature for ZIF-90 [48]. Also, the SEM and TEM analyses displayed a mixed cubic or hexahedral morphology, observable in Figure 1B–D, in agreement with the literature [49].

**Figure 2:**
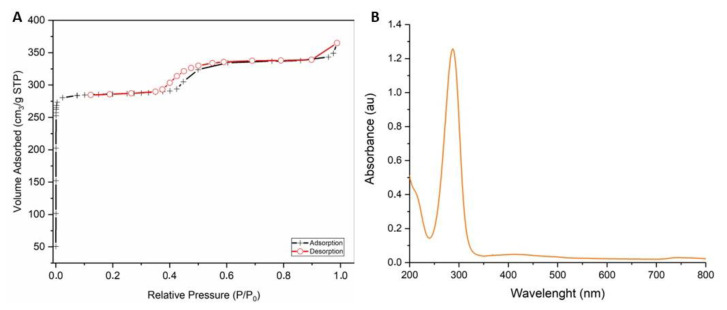
Chemical characterization of ZIF-90. (**A**) N_2_ absorption isotherm at 77 K of ZIF-90 for BET surface area measurement. (**B**) UV–vis spectrum of ZIF-90.

The nitrogen adsorption isotherm of ZIF-90 is shown in Figure 2A, giving an indication of the permanent porosity of the ZIF-90 framework. The curve is a stepped type I isotherm typical of porous compounds containing two distinct families of pores. Micropores are completely filled at pressure values comprising 10^−5^–10^−3^ mbar (0.001 P/P^0^). The small step at higher relative pressure with a hysteresis loop is attributed to the presence of textural mesopores in good agreement with ZIF-90 compounds already reported in other papers [49,50]. The BET (Brunner–Emmett–Teller) surface area is 1195.6 m^2^/g with a micropore volume of 0.42 cm^3^/g. T-plot analysis [51] was also performed in order to calculate the contribution of the micropore surface area over the entire specific surface, resulting in an external surface area of about 32 m^2^/g. The absorbance spectrum in the region of UV–vis for ZIF-90 was obtained (Figure 2B). The spectrum reveals a single peak at 280 nm, which pertains to the imidazole ring.

**Figure 3:**
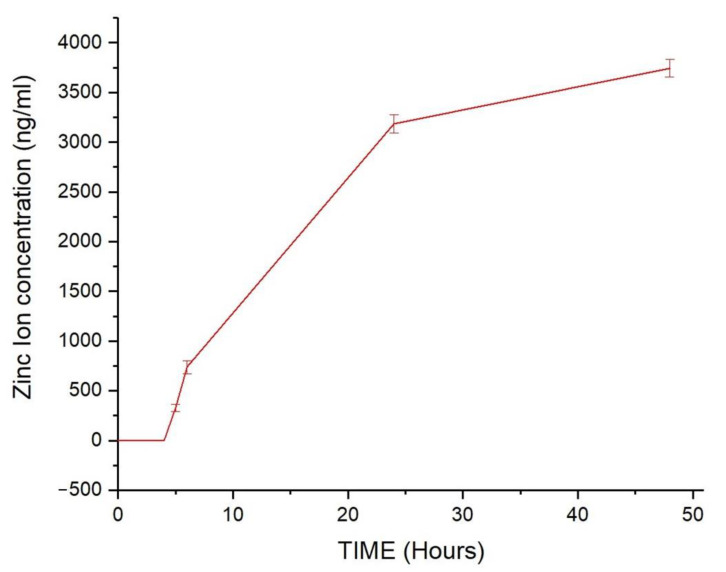
The degradation curve of ZIF-90 measured as zinc ion concentration passed through the dialysis membrane over time.

After chemical characterization, we tested the stability of these nanostructures in a simulated body fluid (SBF). ZIF-90 was dissolved in SBF and put in a dialysis tube for 48 h. Zinc ion concentration, relatable to ZIF-90 dissolution, was measured with ICP at different time points, as it can be observed in Figure 3. ZIF-90 are completely stable for at least 4 h, then they start to dissolve in a logarithmic-like trend, reaching a submaximal zinc concentration after 48 h.

**Figure 4:**
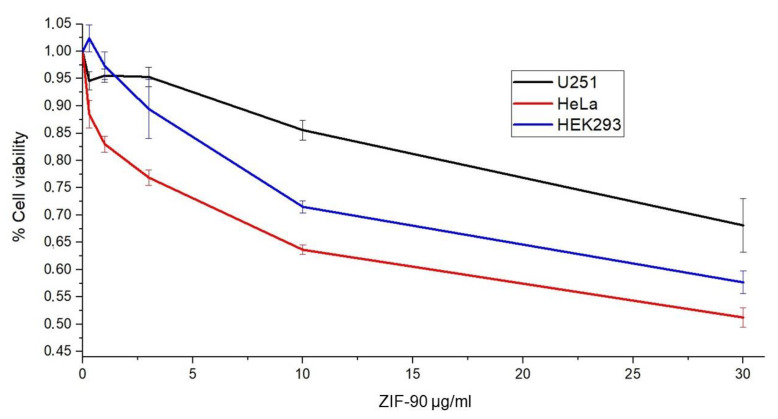
Dose–response curves of ZIF-90 after 48 h of application of U251 (in black) on HeLa (in red) and HEK293 (in blue) cell lines evaluated based on cell viability via MTT assay.

As cellular toxicity is a critical characteristic of a nanocarrier, we estimated it by assessing MTT assays on three different cell lines: U251, HEK293, and HeLa. As displayed in Figure 4, at the highest concentration tested (30 µg/mL), the EC_50_ (effectiveness expressed as % cell viability) was not reached, suggesting low toxicity, in agreement with the literature. 

**Figure 5:**
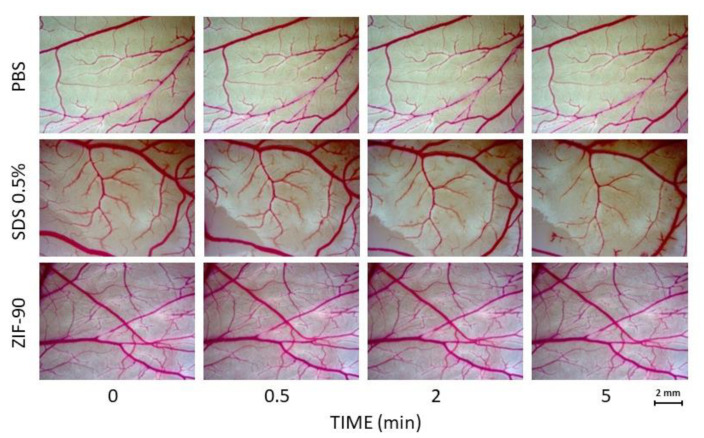
The effects of ZIF-90 on hen chick CAM compared with a negative (PBS) and positive (SDS) control. PBS: phosphate-buffered saline; SDS: sodium dodecyl sulfate.

Another feature that must be taken into account for a nanocarrier is its compatibility in vivo; thus, we evaluated the irritation potential with the Luepke assay [46], using CAMs from chick embryos. Interestingly, ZIF-90 showed the same irritation score as the negative control (Figure 5), meaning 0% probability of irritating the mucous membranes.

**Figure 6:**
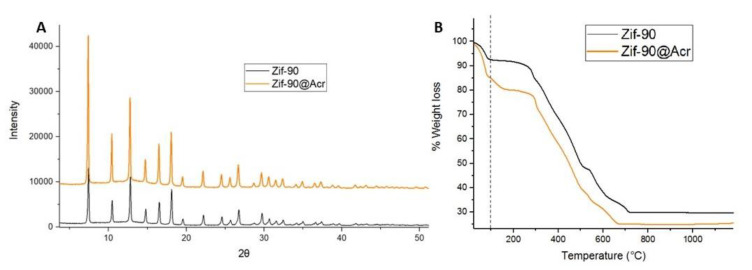
ZIF-90@Acr characterization. (**A**) XPRD of ZIF-90 (in black) compared with ZIF-90@Acr (in orange). (**B**) TG curves of ZIF-90 (in black) compared with ZIF-90@Acr (in orange). The dotted line at 100 °C points to the loss of crystallization water molecules.

To test both the molecule-loading capacity of ZIF-90 and its ability to be internalized in cells and hence release the cargo, ZIF-90 loaded with fluorescent dye AO (ZIF-90@Acr) was synthesized. AO is a fluorescent probe that enables the detection of the entrance of the ZIF@Acr in the cell since it accumulates in acidic compartments, like endosomes. AO is generally used to detect the entry of external substances in these intracellular areas [52]. As it can be observed in Figure 6A, the crystallographic analysis demonstrated that the loading of cargo molecules does not affect synthesis or crystalline structure, as the peaks are not modified. AO’s successful loading was also confirmed by TG analysis, resulting in about 5% loading inside the pores (Figure 6B). It is worth noting that ZIF-90 allowed us to obtain similar internalization results as those reported by us with UiO66 nanoparticles in U251 cells [24].

**Figure 7:**
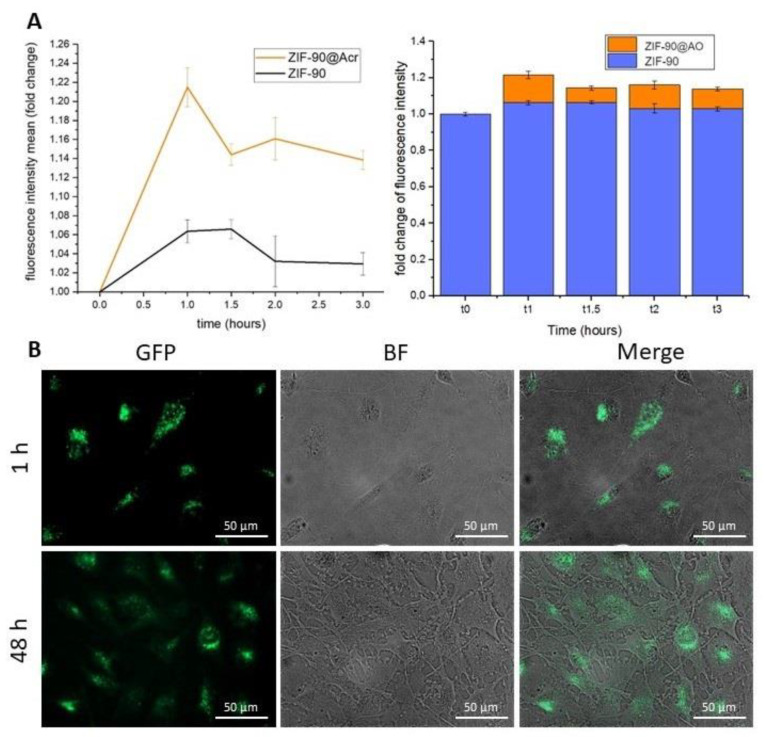
ZIF-90 internalization. (**A**) Fluorescence analysis of U251 cells after ZIF-90 (in black) or ZIF-90@Acr (in orange) treatment. (**B**) Example photos taken in Bright Field (BF) and GFP fluorescence channel (merged on the right) of U251 cells treated with ZIF-90@Acr after 1 or 48 h from administration.

In order to investigate the possibility of ZIF-90 being internalized by cells, ZIF-90 as the negative control and ZIF-90@Acr were dissolved in PBS (1 mg/mL) and dropped on the culture media at 1% in volume. The fluorescence of cells was monitored at different time points through fluorescence microscopy. The plot in Figure 7A shows the increasing fluorescence of cells from the moment of application (T=0) of ZIF-90 or ZIF-90@Acr. As evident, cells started to have a rapid peak of fluorescence after the first hour, and then the fluorescence stabilized to a plateau; this occurred even without AO, as ZIF-90 possesses a slight fluorescence. Interestingly, in Figure 7B is possible to observe that after 1 h ZIF-90@Acr nanoparticles were perfectly recognizable in the intracellular compartment as aggregates; after 48 h, ZIF-90@Acr were still present, but the AO green fluorescence is observable even diffused all over the cell bodies.

**Figure 8:**
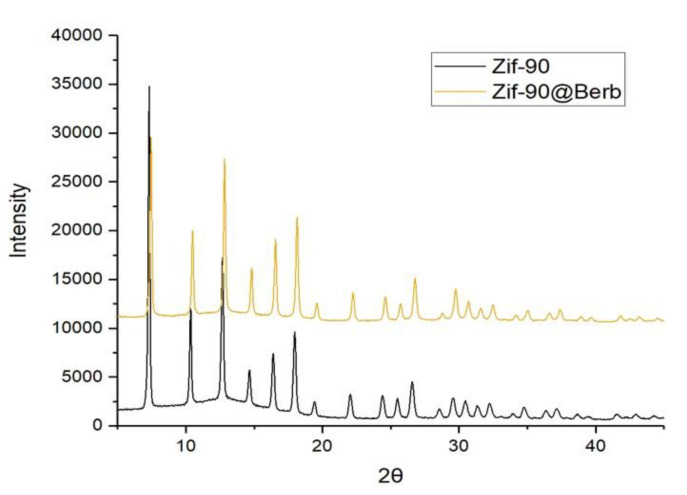
ZIF-90@Berb characterization. XRPD spectra of ZIF-90 (in black) compared with ZIF-90@Berb (in orange).

**Figure 9:**
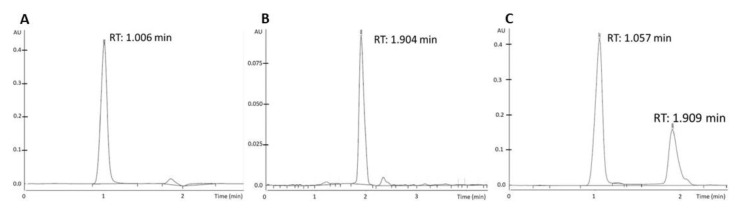
HPLC analysis of ZIF-90@Berb particle preparation. (**A**) Chromatogram of berberine (reference). (**B**) Chromatogram of ZIF-90. (**C**) Chromatogram of ZIF-90@Berb.

In order to involve both ZIF-90 and berberine in glioblastoma treatment, it is of primary importance to demonstrate that they can be combined. Using the same protocol used for the synthesis of ZIF-90@Acr, we succeeded in synthesizing ZIF-90 loaded with berberine (ZIF-90@Berb). As shown in the X-ray analysis in Figure 8, once again berberine did not compromise the synthesis nor crystalline structure of ZIF-90. Using HPLC analysis, we were able to determine the exact ratio between ZIF-90@Berb and berberine mass. The ZIF-90@Berb chromatogram is comparable to the retention time of berberine alone, meaning that the berberine molecule did not undergo structural modifications after ZIF-90@Berb synthesis (Figure 9). After the realization of a calibration line, the berberine in ZIF-90@Berb was quantified at 8.6 ± 1.3 µg/g of powder. Data used for calibration curve construction and berberine quantification can be found in the Supplementary Materials.

## 4. Discussion

MOFs are a class of relatively new materials formed by metallic nuclei and organic linkers. Their versatility and tunable properties (dimension, porosity, reactivity, and toxicity) make them applicable to a very large range of fields. Among all the MOFs, ZIFs have gained attention in the bio and nanomedicine fields [53]. In the field of brain cancer treatment, in particular, ZIFs are promising for their nano dimensions that can penetrate the BBB [13,28,29].

For this work, we chose ZIF-90 as a nanocarrier because of its highly biocompatible metal nuclei (made of zinc) and because of its organic linker (ICA), which makes it suitable for eventual further functionalization, owing to its exposed carbonyl group. The aim of this work is to fully investigate the potentiality of this structure as a nanocarrier.

ZIF-90 synthesis is far faster and greener compared with other MOFs, as crystal precipitation occurs within seconds after the reagents are mixed and the solvents are not toxic. The synthesis is validated via X-ray analysis of dried powder, revealing a very standard and replicable synthetic process. Physico-chemical characterization was completed with high-resolution images using a scansion and transmission electron microscope that displayed cubic or hexahedral morphology, and porosity analysis (BET surface quantification) that revealed high porosity and narrow pore size, in addition to UV–vis spectroscopy measurement. All these data are widely replicated in the literature.

In order to better understand ZIF-90 biocompatibility, we assessed three different assays covering stability in simulated body fluid (SBF), cytopathic effects, and mucous membrane irritation effects. SBF was used as cell culture medium under vigorous and continuous stirring and kept at 37 °C. The stability of ZIF-90 was indirectly measured by the quantification of free zinc ions in the medium. The results indicated that ZIF-90 is very stable in SBF, with no degradation within 4 h and subsequent slight degradation with a plateau at 48 h. Cytotoxic effects were indirectly evaluated by measuring the quantity of MTT converted into formazan crystals by mitochondrial enzyme succinate dehydrogenase, assuming that at saturating conditions the quantity of formazan produced is proportional to the number of cells. The dose–response curves elaborated for HEK293, HeLa, and U251 cells demonstrated high biocompatibility, as the IC_50_ (the inhibitory dose for 50% of the cell population) was not reached for the highest dose tested. Moreover, the Luepke test on chick embryo CAM [46] obtained a score of 0 for ZIF-90, meaning it is a completely harmless material for use on mucous membranes. SDS, a surfactant used as a positive control, obtained the maximum score (score 21, according to literature scheme for irritation testing [46]). 

In order to evaluate ZIF-90 interaction with cells and internalization dynamics, we encapsulated the fluorescent dye AO during ZIF-90 synthesis (ZIF-90@Acr); its loading was proved by TG analysis, which revealed a bigger percentage of weight loss with respect to empty ZIF-90 due to the AO presence. Moreover, X-ray analysis demonstrated that encapsulation of molecules inside ZIF-90 pores does not interfere with crystallinity degree. After cell treatment with ZIF-90@Acr, we traced AO localization over time with a fluorescence microscope. The results revealed a peak of fluorescence, observable as discreet points along the cell body, at 1 h from treatment. This is a good result taking into account that, as previously discussed, ZIF-90 is completely stable in SBF for at least 4 h. A stable fluorescence emission was measured for at least 48 h, even if it is more diffused inside the cell. This is likely due to the release of AO from ZIF-90. Future studies will aim to understand if ZIF-90 is digested by lysosomes.

The final aim of this work was to consider a possible adjuvant drug for therapies against glioblastoma and prove its encapsulation inside ZIF-90. The alkaloid berberine intrigued us, as its anticancer activity against glioblastoma has already been proven [31,54,55]. One pot synthesis of ZIF-90@Berb was analyzed with HPLC in order to determine the exact quantification of berberine loading. Berberine in ZIF-90@Berb was quantified at 8.6 ± 1.3 µg/g of powder. 

## 5. Conclusions

The results obtained suggest that ZIF-90 may represent a promising nanocarrier for directing drugs or active molecules targeting glioblastoma cells. However, the treatment of human glioblastoma represents known and particularly complex difficulties to address, such as limited passage through the BBB or even increased malignancy of the glioblastoma. These are among the most important limitations that have already been encountered in several nanoparticles used in the treatment of glioblastoma [56]. Although some in-depth studies have reported good stability of the porous structure of ZIF-90, we are still far from a definitive treatment. In this work, we demonstrated that ZIF-90 is a biocompatible porous nanocarrier with excellent drug loading and release properties. We successfully produced ZIF-90 particles loaded with berberine and, despite the yield of the loading being so low, the great tunable proprieties of ZIFs give us hope to further improve the encapsulation reaction yield. ZIF-90, therefore, constitutes the right direction toward new therapeutic approaches that provide an answer to these challenges. Interestingly, in this context, the use of ZIF-90 represents the possibility of delivering blockers or modulators of intermediate conductance calcium-activated potassium channels (KCa3.1) involved in human glioblastoma malignancy [57,58,59]. These findings could suggest ZIF-90 as a possible new strategy for brain cancer therapy and also for the study of the physiology of the central nervous system.

## Data Availability

The original contributions presented in the study are included in the article/Supplementary Materials, further inquiries can be directed to the corresponding authors.

## Supplementary Materials

The following supporting information can be downloaded at: https://www.mdpi.com/article/10.3390/pharmaceutics16030414/s1, Figure S1: Calibration curve of standard berberine; Table S1: HPLC analysis of berberine standard solutions; Table S2: Quantification of mass of berberine loaded in the ZIF-90@Berb nanoparticles.
