# Supplementary material for: Synthesis and Characterization of ZIF-90 Nanoparticles as Potential Brain Cancer Therapy
Source: Pharmaceutics. 2024 Mar 18;16(3):414. doi: 10.3390/pharmaceutics16030414 (PMC10975889; doi:10.3390/pharmaceutics16030414)
Supplement: Supplementary file 1 [file pharmaceutics-16-00414-s001.zip › pharmaceutics-2864657-supplementary.pdf]

Supporting Information

Synthesis and characterization of ZIF-90 nanoparticles as potential brain cancer therapy

Lorenzo Monarca [1,2,†], Francesco Ragonese [1,†], Paola Sabbatini [1], Concetta Caglioti [1,2], Matteo Stamegna [1], Federico Palazzetti [1], Paolo Sportoletti [3], Ferdinando Costantino [1,*] and Bernard Fioretti [1,*]

[1] Department of Chemistry, Biology and Biotechnologies, University of Perugia, Via dell'Elce di Sotto 8, 06123 Perugia, Italy

[2] Department of Experimental Medicine, Perugia Medical School, University of Perugia, 06132 Perugia, Italy

[3] Department of Medicine and Surgery, Institute of Hematology, Centro di Ricerca Emato-Oncologica (CREO), University of Perugia, 06129 Perugia, Italy

*Correspondence: ferdinando.costantino@unipg.it (F.C.); bernard.fioretti@unipg.it (B.F.)

† These authors contributed equally to this work.

Quantitative determination of berberine amount in ZIF-90@Berb

Standard solutions for calibration curve have been prepared by dissolving the standard berberine hydrochloride in methanol, to obtain the following concentrations: 2.98 μg/ml; 5.96 μg/ml; 11.9 μg/ml; 23.8 μg/ml; 47.7 μg/ml. The areas of the peak of berberine are reported in Table 1.

Table S1. HPLC analysis of berberine standard solutions.

Berberine Concentration (μg/ml)	Area (AU)
0.04768	40607298
0.02384	20152656
0.01192	10724078
0.00596	6353412
0.00298	3147966

With this data, the calibration curve, depicted in Figure 1, was obtained.

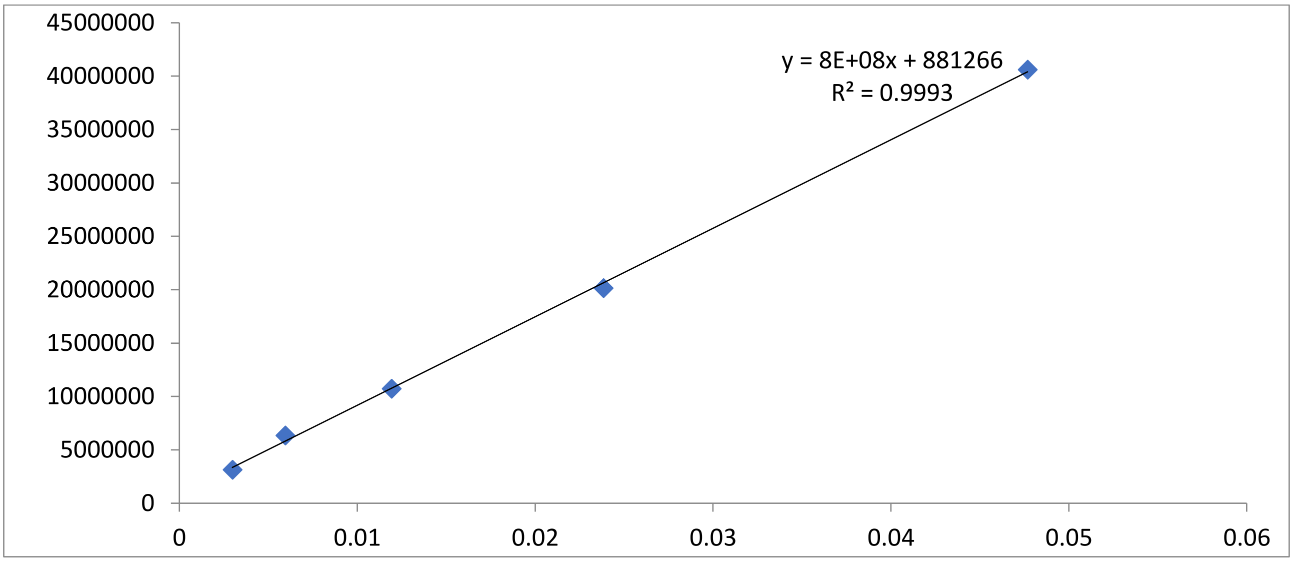

Figure S1. Calibration curve of standard berberine.

As reported in Table 2, three different samples of ZIF-90@Berb of 2.67 g, 1.56 g and 1.78 g respectively were separately dissolved in methanol (10 ml). The three mixtures were vortexed for 15 seconds and centrifugated for 15 minutes at 4.500 g. The supernatants were collected in three 50 ml - Falcon tubes, while the pellets were added of 10 ml of methanol, vortexed for 15 seconds and centrifugated for 15 minutes at 4,500 g again. These second supernatants were jointed to the previously collected ones in the 50 ml-Falcon tubes and vortexed for additional 15 seconds. 20 μl of each solution thus obtained was diluted with 980 μl of methanol and injected to the HPLC column.

Using the calibration curve previously obtained the berberine concentration in the three samples was determined. The mass of berberine loaded in the ZIF-90@Berb was determined as 11.17 μg/g, 6.94 μg/g and 7.69 μg/g respectively. The medium value obtained is therefore of 8.6 ± 1.3 μg each gram of powder.

Table S2. Quantification of mass of berberine loaded in the ZIF-90@Berb nanoparticles.

Mass of ZIF-90@Berb (g)	Area	Berberine concentration (mg/ml)	Mass of berberine loaded in the ZIF-90@Berb (µg/g)
2.67	25633434	0.029832856	11.17335437
1.56	9949661	0.010824429	6.938736487
1.78	12313185	0.013688974	7.690434802
